# Pediatric patients with dog bites presenting to US children’s hospitals

**DOI:** 10.1186/s40621-021-00349-3

**Published:** 2021-09-13

**Authors:** Sriram Ramgopal, Michelle L. Macy

**Affiliations:** 1 Division of Pediatric Emergency Medicine, Department of Pediatrics, Ann & Robert H. Lurie Children’s Hospital of Chicago, Northwestern University Feinberg School of Medicine, 225 E Chicago Ave, Box 62, Chicago, IL 60611 USA; 2 Mary Ann & J. Milburn Smith Child Health Outcomes, Research, and Evaluation Center, Ann & Robert H. Lurie Children’s Hospital of Chicago, Northwestern University Feinberg School of Medicine, Chicago, IL USA

**Keywords:** Bites and stings, Dogs, Accidental injuries, Emergency service, hospital

## Abstract

**Background:**

To characterize pediatric dog bite injuries presenting to US children’s hospitals and identify factors associated with clinically significant injuries.

**Methods:**

We performed a multicenter observational study from 26 pediatric hospitals between July 1, 2010, and June 30, 2020, including patients ≤ 18 years with dog bites, consolidating together encounters from patients with multiple encounters within 30 days as a single episode of care. We characterized diagnoses and procedures performed in these patients. We used generalized linear mixed models to identify factors associated with a composite outcome that we term clinically significant injuries (defined as admission, operating room charge, sedation, fractures/dislocations, intracranial/eye injury, skin/soft tissue infection, or in-hospital mortality).

**Results:**

68,833 episodes were included (median age 6.6 years [interquartile range 3.5–10.4 years], 55.5% male) from 67,781 patients. We identified 16,502 patients (24.0%) with clinically significant injuries, including 6653 (9.7%) admitted, 5080 (7.4%) managed in the operating room, 11,685 (17.0%) requiring sedation, 493 (0.7%) with a skull fracture, 32 (0.0%) with a fracture in the neck or trunk, 389 (0.6%) with a fracture of the upper limb, 51 (0.1%) with a fracture in the lower limb, 15 (0.0%) with dislocations, 66 (0.1%) with an intracranial injury and 164 (0.2%) with an injury to the eyeball, 3708 (5.4%) with skin/soft tissue infections, and 5 (0.0%) with in-hospital mortality. In multivariable analysis, younger age (0–4 years, 5–9 years, and 10–14 years relative to 15–18 years), weekday injuries, and an income in the second and third quartiles (relative to the lowest quartile) had higher odds of clinically significant injuries. Black patients (relative to White), Hispanic/Latino ethnicity, and private insurance status (relative to public insurance) had lower odds of clinically important injuries. When evaluating individual components within the composite outcome, most followed broader trends.

**Conclusion:**

Dog bites are an important mechanism of injury encountered in children’s hospitals. Using a composite outcome measure, we identified younger, White, non-Hispanic children at higher risk of clinically significant injuries. Findings with respect to race and ethnicity and dog bite injuries warrant further investigation. Results can be used to identify populations for targeted prevention efforts to reduce severe dog bite injuries.

**Supplementary Information:**

The online version contains supplementary material available at 10.1186/s40621-021-00349-3.

## Background

Dog bites rank among the most common causes of non-fatal injury among children according to data collected from the National Electronic Injury Surveillance System (Centers for Disease Control and Prevention 2018). While dog bites occur at all ages, these events are particularly common among preadolescent children and account for a higher rate of injury among this group (Weiss et al. 1998; Ramgopal et al. 2018a; Ramgopal and Macy 2021). Dog bites can result in cosmetic and infectious (Drumright et al. 2020) sequelae in many; furthermore, they can occasionally lead to significant craniofacial injury (Khan et al. 2020), fractures, and rarely, death (Sarenbo and Svensson 2020). In developing countries, dog bite injures carry the risk of transmission of the rabies virus, though rates of this disease are low in the USA (Centers for Disease Control and Prevention 2020). Long-term, pediatric dog bite victims are at high risk of psychiatric sequelae (Peters et al. 2004).

Pediatric patients with dog bites frequently present to the emergency department (ED), where they are managed and treated. Most are released to outpatient care (Weiss et al. 1998). However, some patients sustain more significant dog bite injuries requiring care from pediatric subspecialists in children’s hospitals, admission, and/or operative care. One prior report, derived from the Kids' Inpatient Database, suggested approximately 2000 pediatric patients with dog bites were admitted nationally every year, with one-third requiring operative management (McLoughlin et al. 2020). Additional work is required to identify risk factors for the most serious dog bites as these factors can identify targets for public health interventions. Reports from single-center studies, for example, have noted a trend toward younger age and severe injuries with the most substantial complications, such as infection, craniofacial injury, or death (Khan et al. 2020; Saadi et al. 2018). A better understanding of clinically significant outcomes, such as those requiring hospitalization or with complex surgical outcomes, would facilitate future efforts at prevention. In the cases of pediatric traumatic or surgical conditions, children’s hospitals frequently serve as regional referral centers with surgical subspecialty expertise and operative care (Wang et al. 2020). As such, multicenter data from children’s hospitals would allow for the identification of risk factors for clinically important dog bite injuries.

In this investigation, we utilized multicenter data from children’s hospitals to characterize pediatric dog bite injuries. First, we sought to characterize injuries, associated diagnoses, and procedures performed for children with dog bites. Second, we sought to identify risk factors for clinically significant dog bite injuries based on location of injury, complications, and healthcare utilization.

## Methods

### Data source

We performed a retrospective analysis of hospitals within the Pediatric Health Information System (PHIS). PHIS is a multicenter administrative dataset of greater than 40 geographically diverse children’s hospitals within the USA. The Children’s Hospital Association and member hospitals jointly ensure data quality and integrity (Fletcher 2004). This study of existing data was designated as exempt by the Lurie Children’s Hospital Institutional Review Board. For the present analysis, we limited our inclusion to 26 children’s hospitals that contributed data during the years 2010–2020. These included 19 Level I pediatric trauma centers, 6 Level II pediatric trauma centers, and one non-trauma center. Twenty-five of the institutions were freestanding children’s hospitals. Hospital administrative data are collected by each hospital and submitted to PHIS. Practices for collecting race/ethnicity may vary hospital to hospital.

### Inclusion

We identified encounters ≤ 18 years of age for inclusion using diagnosis codes for dog bites during the time period of July 1, 2010, to June 30, 2020. We acquired cases using International Classification of Disease (ICD), ninth and tenth revisions. We used the ICD-9 external cause of injury code E906.0 (dog bite) for encounters prior to October 31, 2015, and the ICD-10 code W54.0XXA (dog bites, initial encounter) following this date. We excluded encounters listed as clinic visits. For patients with multiple encounters for dog bites during the inclusion period, we considered all encounters within a 30-day time interval as a single episode of care. Patients with dog bite encounters separated by more than 30 days were considered as distinct episodes of care.

### Data acquisition

We acquired the following data with respect to each episode of care. Demographic data included age (in categories of 0–4 years, 5–9 years, 10–14 years, and 15–18 years), sex, race (characterized using groupings provided in the PHIS dataset), ethnicity (Hispanic or non-Hispanic), payor type (classified into groups of public, private, and other/unknown), median household income of the child’s ZIP code, Census region, day of week, and season. Treatment data collected included ED care (defined as an ED charge), ambulatory surgery visit, transfer from another acute care or inpatient facility, and disposition (classified as admitted, discharged, and transferred to another institution), operative care (defined as an operating room [OR] charge), intensive care unit admission, and in-hospital mortality. We included admission under observation or inpatient status on the basis of previous work suggesting that these types of encounters were similar with respect to resource utilization (Fieldston et al. 2013). We additionally reported the number of patients with a concomitant diagnosis of skin/soft tissue infection or use of oral or intravenous antibiotics (given during the hospital stay) during their episode. PHIS does not contain data on the outpatient use of antibiotics (i.e., as a prescription). We identified patients who received rabies prophylaxis, either as a rabies vaccine or rabies immune globulin. We identified skin/soft tissue infection via use of ICD-9 diagnosis code 680–686 and ICD-10 diagnosis code L00–L08.

### Diagnoses

We assessed for concomitant traumatic diagnoses using groupings within ICD-9 categories. For ICD-10 codes, we converted these to ICD-9 codes as an initial step using generalized equivalence mappings (GEMS). GEMS are a tool provided by the Centers for Medicare and Medicaid services that provide a bidirectional crosswalk between ICD-9 and ICD-10 codes (ICD-10 | CMS 2020). We grouped traumatic diagnostic codes with ICD-9 codes (Additional file 1: Table S1). We further subdivided skull fractures into the following: skull vault, skull base, facial bones, other/unspecified, and multiple fractures involving skull or face with other bones. Fractures of the neck and trunk were characterized as fracture of the vertebral column without spinal cord injury, fracture of the vertebral column with spinal cord injury, fracture of the rib, sternum, larynx and trachea, fracture of the pelvis, and unspecified. We subdivided open wounds of the head, neck, and trunk into subgroups of ocular adnexa, eyeball, ear, head, neck, chest wall, back, buttock, genital organs, and unspecified.

### Procedures, specialty, and sedation

We categorized billed procedures using ICD-9 codes. As with diagnoses, we crosswalked ICD-10 to ICD-9 procedure codes as an initial step. We identified episodes of care where any of the following were performed (Additional file 2: Table S2). We additionally acquired the number of patients who had at least one procedure performed and subspecialties of providers performing types of procedures. We characterized sedation as provision of any of the following: propofol, ketamine, etomidate, thiopental, sevoflurane, desflurane, isoflurane, and nitrous oxide.

### Outcome

Our outcome of interest was clinically significant dog bites, characterized by indicators of higher morbidity based on the care provided or injuries sustained. We defined this as any of the following: hospital admission, operating room charge or use of sedation medications, fractures, and dislocations (Khan et al. 2020; Saadi et al. 2018), intracranial injury and skull fractures (Steen et al. 2015), injuries to the eyeball (Hurst et al. 2020), skin/soft tissue infection (Drumright et al. 2020), and in-hospital mortality.

### Analysis

We assessed the proportion of ED encounters for dog bites over time relative to all ED encounters, prior to consolidating patient encounters into 30-day episodes of care and assessed for a correlation between the monthly proportion of encounters for dog over time using the Spearman rank correlation test. As previous data have suggested a rise in the rate of dog bites relative to other encounters in the setting of the COVID-19 pandemic likely due to a decrease in ED encounters for other reasons (Dixon and Mistry 2020), we limited this analysis to the years 2010–2019.

Following our consolidation of encounters within 30-day episodes of care, we characterized them based on ED utilization, inpatient stay (with and without intensive care unit use), ambulatory surgery status, sedation, and OR utilization and reported proportions of patients in each group with a skin/soft tissue infection diagnosis. We reported demographics and treatment factors, reporting raw numbers with proportions. For all dog bites, we reported procedures performed by category and most common subspecialties of physicians performing procedures by episode. We reported proportions of episodes having a concomitant diagnosis of skin/soft tissue infection, the proportion given oral or intravenous antibiotics, and the most frequently used antibiotics.

We performed logistic regression to identify factors associated with clinically significant dog bites. We considered demographics of age group, sex, race, ethnicity, season, and day of week, payor type, and median household income by patient ZIP code as candidate variables. Despite some data suggesting differential rates in pet ownership by race and ethnicity (Loder 2019) and potential collinearity with these variables, we considered these in our logistic regression modeling given that a number of dog bite injuries (between 20 and 50% in some studies) (Loder 2019; Bykowski et al. 2017) do not occur in a patient’s home, and because of the complex association between racial and ethnic factors with pet ownership among households with children (Strochak et al. 2018).

Median household income was categorized by quartile. For modeling, we collapsed race into categories of White, Black and other, and day of week into weekday and weekend (Saturday and Sunday). We performed multiple imputation using chained equation for missing values of sex, ethnicity, and median income by ZIP code (van Buuren and Groothuis-Oudshoorn 2011). We performed univariate and multivariable analysis using all variables, reporting odds ratios with 95% confidence intervals using a generalized linear mixed model with a binary outcome and considering each hospital as a random effect. Analyses were performed using the *lme4* (Bates et al. 2015) package in R version 3.6.3 (R Foundation for Statistical Computing, Vienna, Austria).

### Additional analyses

As a sensitivity analysis, we repeated our analysis for clinically important dog bite injuries restricted to patients 0–4 years of age, since some individual outcomes were less frequent among older patients. As an exploratory analysis, we created univariable and multivariable models for the following individual outcomes within our composite measure: (1) admission, (2) OR charge or sedation, (3) dislocations and fractures (excluding skull fractures), (4) intracranial injury, skull fractures, and eyeball injuries, (5) skin/soft tissue infection, and (6) in-hospital mortality. Because of few cases of in-hospital mortality, we only performed univariable analyses for this outcome.

## Results

### Inclusion

Among the 26 included hospitals, 72,731 encounters were identified in the query. Prior to 2020, dog bites accounted for 3.0 per 1000 ED encounters. The rate of ED encounters for dog bites relative to all ED encounters was not significantly changed over time (Spearman’s rho = 0.21, *p* = 0.56). After applying exclusions to consolidate encounters into episodes of care, 68,833 were included (Additional file 3: Figure). There were 67,781 unique patients in the included dataset. Most patients (66,767, 98.5%) had a single encounter. Among patients with multiple episodes of care (i.e., having encounters occurring at time intervals greater than 30 days apart), 980 (1.4%) had two, 31 (< 0.1%) had three, 2 (< 0.1%) had four, and 1 (< 0.1%) had five. The median episode age was 6.6 years (interquartile range 3.5–10.4 years), and 55.5% were male. Dog bites presented most frequently among patients 0–4 years of age, among White patients, and on Saturday or Sunday. Dog bites demonstrated a slight spring/summer predominance (Table 1).

**Table 1:** Demographics and selected treatment factors of encounters with dog bites

Variable	Encounters with dog bites
*Demographics*	N (%) or median (IQR) *N* = 68,833
Age	
0–4 years	26,162 (38.0)
5–9 years	23,719 (34.5)
10–14 years	14,445 (21.0)
15–18 years	4507 (6.6)
Sex	
Male	38,182 (55.5)
Female	30,629 (44.5)
Unknown	22 (0.0)
Race	
White	44,081 (64.0)
Black	12,132 (17.6)
Asian	1125 (1.6)
Native American	214 (0.3)
Pacific Islander	118 (0.2)
Other or more than one	8575 (12.5)
Missing	2588 (3.8)
Hispanic ethnicity	
Hispanic or Latino	17,395 (25.3)
Not Hispanic or Latino	44,374 (64.5)
Unknown	7064 (10.3)
Payor type	
Private	25,463 (37.0)
Public	36,649 (53.2)
Other/unknown	6721 (9.8)
Day of week	
Sunday	12,242 (17.8)
Monday	9542 (13.9)
Tuesday	8860 (12.9)
Wednesday	8527 (12.4)
Thursday	8682 (12.6)
Friday	9204 (13.4)
Saturday	11,776 (17.1)
Season	
Winter	15,037 (21.8)
Spring	20,003 (29.1)
Summer	18,657 (27.1)
Fall	15,136 (22.0)
Census region	
Northeast	6643 (9.7)
Midwest	14,376 (20.9)
South	29,519 (42.9)
West	18,295 (26.6)
Median household income of ZIP Code, dollars	41,344 (32,593–54,477)
Transferred from another facility	3097 (4.5)
Had an ED encounter	63,761 (92.6)
*Treatment characteristics*	
Admitted	6653 (9.7)
Transferred to another facility	453 (0.7)
Need for operating room	5080 (7.4)
Intensive care unit admission	242 (0.4)
In-hospital mortality	5 (0.0)

*IQR* interquartile range

### Clinically significant dog bites

Overall, 16,502 (24.0%) had clinically significant dog bites. Among included episodes, 6653 (9.7%) of were admitted, 5080 (7.4%) received management in the OR, and 11,685 (17.0%) received sedation. 493 (0.7%) had a skull fracture, 32 (0.0%) had a fracture in the neck or trunk, 389 (0.6%) had a fracture of the upper limb, 51 (0.1%) had a fracture in the lower limb, and 15 (0.0%) had a dislocation. 66 (0.1%) had an intracranial injury, and 164 (0.2%) had injury to the eyeball. Skin/soft tissue infections were identified in 3708 (5.4%). Five (0.0%) episodes of care had an outcome of in-hospital mortality.

### Patient flow pathways (Additional file 4: Table S3)

Among episodes with only a single encounter within a 30-day period (*n* = 66,752, 97.0%), most (79.1%) were limited to the ED alone and did not require admission or use of the OR/sedation. A smaller proportion of patients were admitted, with or without use of the OR, represented direct admissions without an ED encounter, or were ambulatory surgery visits. A diagnosis of skin/soft tissue infection was made frequently among admitted patients. Among episodes of care with multiple encounters within a 30-day range, overall flow pathways were similar, with many (45.4%) limited to ED encounters only.

### Diagnoses (Table 2)

**Table 2:** Traumatic diagnoses among dog bite victims

Traumatic diagnoses	Overall number (%) (*N* = 68,833)
Skull fracture	493 (0.7)
Skull vault	82 (0.1)
Skull base	129 (0.2)
Facial bones	334 (0.5)
Other or unspecified	10 (0.0)
Multiple	0 (0.0)
Fracture of neck and trunk	32 (0.0)
Vertebral column without spinal cord	9 (0.0)
Vertebral column with spinal cord	0 (0.0)
Ribs, sternum, larynx, trachea	22 (0.0)
Pelvis	3 (0.0)
Unspecified	0 (0.0)
Fracture of upper limb	389 (0.6)
Fracture of lower limb	51 (0.1)
Dislocation	15 (0.0)
Sprains and strains of joints	63 (0.1)
Intracranial injury	66 (0.1)
Thorax, abdomen and pelvis	61 (0.1)
Open wound of head, neck and trunk	40,801 (59.3)
Ocular adnexa	4879 (7.1)
Eyeball	164 (0.2)
Ear	2999 (4.4)
Other head	33,877 (49.2)
Neck	1006 (1.5)
Chest	545 (0.8)
Back	521 (0.8)
Buttocks	666 (1.0)
Genital organs	343 (0.5)
Unspecified	1339 (1.9)
Open wound of upper limb	13,796 (20.0)
Open would of lower limb	8218 (11.9)
Injury to blood vessels	60 (0.1)

Some encounters may not have had a traumatic diagnosis captured into a group, and others may have had more than one traumatic diagnosis

Most (59.3%) episodes had open injuries of the head and neck region, followed by injuries of the upper (20.0%) and lower (11.9%) extremities. Among patients with skull fractures, fractures of the facial bones were the most common, present in 67.7%.

### Procedures performed (Table 3)

**Table 3:** Procedures performed

Procedure grouping	All encounters with a billed procedure (%) (*N* = 15,445)
Nervous system	231 (1.5)
Eye	2110 (13.7)
Ear	976 (6.3)
Nose, mouth, pharynx	3957 (25.6)
Respiratory	106 (0.7)
Digestive	209 (0.6)
Genitourinary	99 (0.6)
Musculoskeletal	2066 (13.4)
Integumentary	10,098 (65.4)

Some encounters may not have had a procedure diagnosis captured into a group, and others may have had more than one procedure

Overall, 22.4% episodes of care had a billed procedure, with the most common representing integumentary repairs (65.4%). When evaluating by subspecialty, episodes had procedures performed by physicians in the following proportions: emergency medicine (43.3%), plastic surgery (13.7%), general pediatrics (6.7%), general surgery (4.3%), ophthalmology (5.2%), otorhinolaryngology (3.9%), family practice (2.8%), orthopedic surgery (2.4%), sports medicine (2.3%), gynecology (1.3%), oral maxillofacial surgery (1.1%), neurosurgery (0.5%), and dentistry (0.5%).

### Infection, use of antimicrobials, and rabies prophylaxis

Among episodes with dog bites, 30,890 (44.9%) of all episodes were ordered in-hospital antibiotics and 3708 (5.4%) had a skin/soft tissue diagnosis code associated with their episode. The most commonly used antibiotics were amoxicillin–clavulanate (59.5%), ampicillin–sulbactam (34.6%), clindamycin (10.1%), cefazolin (6.0%), sulfamethoxazole/trimethoprim (3.2%), and piperacillin–tazobactam (2.4%). Rabies prophylaxis was provided in 3,678 patients (5.3%).

### Factors associated with clinically important injury (Table 4)

**Table 4:** Factors associated with clinically significant dog bite injuries

Variable	Not clinically significant (*N* = 52,331)	Clinically significant (*N* = 16,502)	Univariable odds of clinically significant injury		Multivariable odds of clinically significant injury aOR (95% CI)	
	*N* (%)	*N* (%)	OR (95% CI)	*p*	aOR (95% CI)	*p*
Age						
0–4 years	17,626 (33.7)	8536 (51.7)	3.68 (3.35–4.05)	< 0.001	3.21 (2.92–3.54)	< 0.001
5–9 years	18,495 (35.3)	5224 (31.7)	2.16 (1.96–2.38)	< 0.001	2.03 (1.85–2.24)	< 0.001
10–14 years	12,235 (23.4)	2210 (13.4)	1.36 (1.23–1.51)	< 0.001	1.32 (1.19–1.46)	< 0.001
15–18 years	3975 (7.6)	532 (3.2)	Ref.	–	Ref.	–
Male sex	29,350 (56.1)	8845 (53.6)	0.91 (0.88–0.95)	< 0.001	0.96 (0.93–1.00)	0.048
Race						
White	33,306 (63.6)	12,480 (75.6)	Ref.	–	Ref.	–
Black	10,439 (19.9)	2095 (12.7)	0.48 (0.46–0.51)	< 0.001	0.49 (0.46–0.52)	< 0.001
Other	8586 (16.4)	1927 (11.7)	0.60 (0.57–0.64)	< 0.001	0.73 (0.68–0.77)	< 0.001
Hispanic or Latino	15,817 (30.2)	3354 (20.3)	0.54 (0.51–0.56)	< 0.001	0.51 (0.48–0.54)	< 0.001
Payor type						
Public	28,045 (53.6)	8604 (52.1)	Ref.	–	Ref.	–
Private	19,121 (36.5)	6342 (38.4)	1.09 (1.05–1.14)	< 0.001	0.80 (0.77–0.84)	< 0.001
Other/unknown	5165 (9.9)	1556 (9.4)	0.87 (0.82–0.93)	< 0.001	0.80 (0.75–0.86)	< 0.001
Weekday encounter	33,926 (64.8)	10,889 (66.0)	1.05 (1.01–1.09)	0.011	1.06 (1.02–1.10)	0.002
Season						
Winter	11,375 (21.7)	3662 (22.2)	Ref.	–	Ref.	–
Spring	15,297 (29.2)	4706 (28.5)	0.93 (0.85–1.01)	0.212	0.99 (0.94–1.04)	0.698
Summer	14,281 (27.3)	4376 (26.5)	0.96 (0.88–1.04)	0.290	0.98 (0.93–1.04)	0.558
Fall	11,378 (21.7)	3758 (22.8)	1.00 (0.92–1.10)	0.279	1.01 (0.96–1.07)	0.663
Median household income, quartile						
First	13,712 (26.2)	3543 (21.5)	Ref.	–	Ref.	–
Second	12,828 (24.5)	4410 (26.7)	1.36 (1.29–1.43)	< 0.001	1.14 (1.08–1.21)	< 0.001
Third	12,846 (24.5)	4334 (26.3)	1.46 (1.39–1.55)	< 0.001	1.15 (1.09–1.22)	< 0.001
Fourth	12,945 (24.7)	4215 (25.5)	1.41 (1.34–1.50)	< 0.001	1.01 (0.95–1.07)	0.856

*OR* odds ratio, *aOR* adjusted odds ratio, *CI* confidence interval

In univariable analysis, age, race, ethnicity, payor type, and median household income were associated with dog bite injuries. In multivariable analysis, younger age, weekday status, and the second and third median household incomes by ZIP code (relative to the lowest quartile) were positively associated with clinically important injuries. Black and Other race (compared to White), private and other/unknown payor types (compared to public), and Hispanic/Latino ethnicity (compared to non-Hispanic/Latino ethnicity) had a lower adjusted odds of clinically important injuries.

### Additional analyses

Findings from a sensitivity analysis limited only to patients 0–4 years of age use were similar to the primary analysis (Additional file 5: Table S4). When evaluating individual outcomes within the composite outcome, most individual outcomes followed broader trends (Additional file 6: Table S5, Additional file 7: Table S6, Additional file 8: Table S7, Additional file 9: Table S8, Additional file 10: Table S9 and Additional file 11: Table S10). Fractures had a lower odds of occurring in younger patients, and low counts limited the interpretability of odds ratios for in-hospital mortality.

## Discussion

Using a large administrative dataset, we reported the experience among children’s hospital encounters with dog bites. Using a composite outcome, 24% of patients met our criteria for clinically important injury. We found that victims with dog bites required hospitalization in 10% and operative management in 7%. In multivariable analysis, younger age, White race, non-Hispanic ethnicity, and higher-income levels were associated with clinically significant injury.

Several studies have described epidemiologic trends with respect to dog bite injuries (Weiss et al. 1998; Ramgopal et al. 2018a; Ramgopal and Macy 2021). The results from this study expand on those findings in important ways. First, we evaluated outcomes of dog bite injuries encountered from a large database of children’s hospitals, most of which serve as trauma centers and referral centers. These hospitals encounter a higher proportion of dog bites requiring admission or operative intervention and allow for the characterization of uncommon injury types. Second, we developed a composite measure for clinically important bites to guide future research using data which can be ascertained from administrative data or the electronic medical record. Third, in our multivariable analyses, we identified populations at greatest risk of clinically important dog bites, which carry the greatest patient morbidity and require the greatest resource utilization. These findings included associations with race and ethnicity that persisted in most analyses when investigating individual components of the outcome.

Our study compares to prior work on pediatric dog bite injuries in several ways. While the majority of dog bite episodes in our study had open wounds without more substantial traumatic injury, a smaller percentage sustained fractures, including of the craniofacial region and long bones, genitourinary trauma, and intracranial hemorrhage. Prior work from one single-center study has suggested that a low proportion of pediatric patients with dog bites (1.5%) sustained traumatic fractures to the scalp and face (Saadi et al. 2018). An analysis of 182 patients with craniofacial injuries, of which 19 had fractures, suggested that younger age and female patient sex were at higher risk of these conditions (Khan et al. 2020). Our findings with respect to dog bite injuries are also higher than an analysis using the Kids’ Inpatient Database for the years 2006, 2009, and 2012, which reported that approximately 1/3 of admitted patients with dog bites required an operative intervention, with the highest proportion having open wounds of the head and neck, followed by open wounds of the extremities (McLoughlin et al. 2020). We found that a slightly greater proportion (approximately half of admitted patients) required management in the OR. This may be due to our inclusion of children’s hospitals, most of which were classified as Level 1 or 2 trauma centers. This may yield a sample biased toward patients with severe injuries,

We identified associations with age, race, and ethnicity with clinically important dog bite injuries in multivariable analysis. Among non-fatal dog bite injuries for the years 2010–2019 within the Web-based Injury Statistics Query and Reporting System (WISQARS), the crude rate of dog bites among patients 0–19 years of age was 156.6 per 100,000 individuals (Centers for Disease Control and Prevention 2021). 4.1 children with dog bites per 100,000 population, (or 2.5% of patients with dog bites), required hospital admission or transfer to another hospital. The proportion needing admission/transfer within the WISQARS dataset, similar to another reported rate of 2.7% among patients of all ages using a nationally representative dataset of US ED encounters (Ramgopal and Macy 2021), is lower than the described proportion of admission in our study (9.7%) and likely relates to our hospital inclusion.

Our findings with respect to clinically significant outcomes with age and race appear similar to the WISQARS dataset and support the generalizability of these findings. A higher proportion of bite victims having a disposition of admission/transfer was identified among those of younger age (7.6 needing admission/transfer per 10,000 among 0–4 years compared to 1.9 needing admission/transfer among 15–19 years, compared to 176.4 dog bite encounters per 100,000 among 0–4 years and 112.6 dog bite encounters per 100,000 among 15–19 years). Similarly, a differential in admission/transfer was noted among White patients (4.3 per 10,000 White patients and 1.9 per 10,000 Black patients) relative to dog bite encounters overall (153.2 dog bites per 100,000 White patients and 106.3 bites per 100,000 Black patients), though WISQARs carries limitations with the reporting of race and ethnicity data (Centers for Disease Control and Prevention 2021).

Other findings in this study corroborate with previously published reports on dog bites. Our finding with respect to younger age likely relates to their immature behavior and shorter height (Ramgopal et al. 2018b). Similar to prior literature, we identified a higher rate of injuries among children 0–9 years of age (Ramgopal et al. 2018b; Basco et al. 2020) and among males (Ramgopal et al. 2018b). A high proportion of patients had head and neck injuries (Hurst et al. 2020; Ramgopal et al. 2018b; Zangari et al. 2020). In addition to traumatic injuries, we identified that 5.4% of episodes for dog bites had infectious complications, which corresponds to research reporting a rate of infection of 2.5% among adult and pediatric victims of dog bites in the ED (Dire et al. 1994). This proportion is lower than other reported evidence suggesting higher rates of wound infection (10–15%) (American Academy of Pediatrics 2018), which may relate to patients seeking care at alternative sites for these infections (such as primary care offices), or potential under-coding of infections when applied as a secondary diagnosis.

Our findings with respect to race and ethnicity are notable. We found that White children also had disproportionately higher rates of clinically significant injuries. Dog bites have previously been described to occur more frequently among White non-Hispanic patients relative to patients of other races (Ramgopal and Macy 2021; Loder 2019; Centers for Disease Control and Prevention 2021), a finding which may be associated with higher reported rates of pet ownership among White households (American Veterinary Medical Association 2017). Our findings are also aligned with racial/ethnic patterns of dog bite injuries in the WISQARS dataset (Centers for Disease Control and Prevention 2021) and prior case–control research (Parent et al. 2021; Rhea et al. 2014). The reasons for this finding are likely multifactorial and warrant further investigation through an adequately powered prospective study designed to capture data about dog ownership, breed, circumstances leading up to the injury, and medical decision-making. A child’s racial/ethnic background may lead to differential exposure to larger, more aggressive, or temperamental breeds, which cannot be ascertained from administrative datasets and additionally confound our results. For example, in one prior case–control study of craniofacial dog bites in children, the odds ratio of craniofacial fractures from dog bites for White patients increased after adjustment for large dog size (unadjusted odds ratio 7.3, 95% confidence interval 1.6–16.7; adjusted odds ratio 11.2, 95% confidence interval 2.2–56.7) (Parent et al. 2021). Different practice patterns may exist within hospitals. Practice patterns may be associated with predominant demographic characteristics in the patient population or payer mix.

We identified that the second and third income quartiles (compared to the lowest income level) and that public insurance status (relative to private insurance status) had a higher adjusted odds of clinically significant injuries. Differential care-seeking behaviors and caregiver expectations may play a role in these findings. Lower-income patients may seek care in ED more frequently for minor injuries relative to those with higher incomes, for reasons related to medical literacy, access to care, and insurance coverage (Kutner et al. 2003); as such, this group may present with a higher proportion of lower-acuity injuries overall relative to the middle two quartiles. Nonetheless our findings compare with data suggesting that dog bite injuries (regardless of severity) are associated with lower socioeconomic status (Loder 2019; Rhea et al. 2014; Tuckel and Milczarski 2020). Income may serve as a proxy for factors which correlate with dog bite injury. For example, income correlates with rurality and may also serve as a proxy for pet ownership or family size (where a larger family may prevent closer supervision of individual children) (Loder 2019; Tuckel and Milczarski 2020). Additionally, dog breed factors may play a role in explaining our findings in relation to income and insurance status. In one study evaluating dog bite injuries among adults presenting to a Level 1 trauma center, there was an association between dog breed and income. Pit bull bites were more common among patients with lower-incomes than victims bitten by other breeds (Lee et al. 2019). Income, healthcare access, and/or rurality may also correlate with treatment decisions in the hospital, including in the determination of hospital disposition. Patients transferred into a children’s hospital may be more likely to be admitted or undergo procedures. Further evaluation is required to better characterize the influence of these factors on dog bite injuries and prevention strategies.

In addition to identifying targeted populations at highest risk of clinically significant injuries, our findings require further research. While other large datasets appear to corroborate our findings (Centers for Disease Control and Prevention 2021), additional work is required to identify whether they are generalizable with respect to the composite measure overall (including characterizing the indication for hospital admission) and with respect to its individual findings. Our associations with respect to race, ethnicity, and income require additional investigation to better characterize the severity of specific dog bites injuries and identify at-risk populations. As retrospective or registry-based studies of dog bite injuries are frequently limited at characterizing the specific nature of injuries or their precipitating causes (such as dog breed and location of injury, such as if the patient was bitten by a family dog, a neighbor’s pet, or a stray animal) (Ramgopal et al. 2018a; Bykowski et al. 2017; Tuckel and Milczarski 2020), prospective work will likely be required to characterize injuries and elucidate specific risk factors.

While further research is required to understand etiologies of demographic differences in our study, our findings, nonetheless, identify target groups for educational preventive interventions. Efforts could focus on the highest risk populations (such as White non-Hispanic families with young children). The American Veterinary Medical Association’s Task Force on Canine Aggression and Human-Canine Interactions promotes a multifaceted approach toward dog bite prevention, including the control of free roaming animals, dog licensure, and legislative approaches (American Veterinary Medical Association 2001). Educational interventions could be disseminated in primary care offices, shelters and kennels, pet supply stores, and veterinarian offices and include recommendations to avoid the acquisition of a large dog in families with young children, adequately separate children and dogs, and emphasize the need for close supervision. One meta-analysis of nine studies concluded that cognitive and behavioral interventions had a moderate effect on improving children’s knowledge, though overall evidence was poor (Shen et al. 2016). An ED-based study using a video intervention, for example, demonstrated improved knowledge among children when comparing pre- and post-tests, though younger age (a particularly high-risk group in this study) was associated with lower passing scores (Dixon et al. 2013). The limitations with educating younger children underscores the importance of careful parental supervision.

Our findings are subject to limitations. The PHIS dataset may be subject to limitations in accuracy and errors in coding. We included patients using ICD codes, and potentially injuries which did not have a cause of injury diagnosis code assigned (i.e., and were only for ‘laceration’) would have been missed by this method. A similar limitation lies with the identification of skin/soft tissue infections, which happens several days after a dog bite injury: Some encounters for dog bites may be related to delayed infectious complications following the initial traumatic injury. Given the data source, our findings are likely skewed toward more severe injuries. Procedures that were performed but not billed will not be present in PHIS; this may be the case in some teaching hospitals where a subspecialty resident performs the procedures but does not bill for it. Prescription antibiotics are not contained in the PHIS dataset. Despite these limitations, the findings from this study represent the collective experience from many pediatric hospital and provide longitudinal data with respect to this frequently encountered pediatric injury.

## Conclusion

In this multicenter analysis of pediatric dog bites in children’s hospitals, we found that dog bites occurred in 0.3% of ED encounters. Overall, 24.0% of children had clinically significant dog bites. Younger age, White race, and non-Hispanic ethnicity are associated with a higher rate of clinically significant serious injury. Our findings with respect to race and ethnicity and dog bite injuries warrant further investigation. These results can be used to identify populations for targeted prevention efforts to reduce severe dog bite injuries.

## Supplementary Information

**Additional file 1: Table S1.** Diagnoses, by groupings of International Classification of Disease, 9th revision (ICD-9) codes.

**Additional file 2: Table S2.** Procedures, by groupings of International Classification of Disease, 9th revision (ICD-9) codes.

**Additional file 3: Figure.** Patient inclusion.

**Additional file 4: Table S3.** Patient encounter pathways.

**Additional file 5: Table S4.** Factors associated with clinically significant dog bite injuries as a sensitivity analysis when limited to encounters of patients ≤ 4 years of age (n = 26,162).

**Additional file 6: Table S5.** Exploratory analysis of factors associated with clinically important outcomes, analyzed by individual outcome measures; outcome 1: admission.

**Additional file 7: Table S6.** Exploratory analysis of factors associated with clinically important outcomes, analyzed by individual outcome measures; outcome 2: sedation or operating room charge.

**Additional file 8: Table S7.** Exploratory analysis of factors associated with clinically important outcomes, analyzed by individual outcome measures; outcome 3: fractures (excluding skull fractures).

**Additional file 9: Table S8.** Exploratory analysis of factors associated with clinically important outcomes, analyzed by individual outcome measures; outcome 4: skull fractures, intracranial/orbital injury.

**Additional file 10: Table S9.** Exploratory analysis of factors associated with clinically important outcomes, analyzed by individual outcome measures; outcome 5: skin/soft tissue infection.

**Additional file 11: Table S10.** Exploratory analysis of factors associated with clinically important outcomes, analyzed by individual outcome measures; outcome 6: death.

## Data Availability

The data that support the findings of this study are available from The Children’s Hospital Association, but restrictions apply to the availability of these data, which were used under license for the current study, and so are not publicly available. Data are, however, available from the authors upon reasonable request and with permission of The Children’s Hospital Association.

## Abbreviations

PHIS: Pediatric health information system
ICD: International classification of disease
ED: Emergency department
