# Supplementary material for: Pediatric patients with dog bites presenting to US children’s hospitals
Source: Inj Epidemiol. 2021 Sep 13;8:55. doi: 10.1186/s40621-021-00349-3 (PMC8436008; doi:10.1186/s40621-021-00349-3)
Supplement: Supplementary file 1 — Additional file 1: Table S1. Diagnoses, by groupings of International Classification of Disease, 9th revision (ICD-9) codes. [file 40621_2021_349_MOESM1_ESM.docx]

**Additional file 1: Table S1.** Diagnoses, by groupings of International Classification of Disease, 9^th^ revision (ICD-9) codes.

| **Traumatic diagnoses** | **ICD-9 diagnoses** |
| --- | --- |
| Skull fracture | 800-804 |
| Skull vault | 800 |
| Skull base | 801 |
| Facial bones | 802 |
| Other or unspecified | 803 |
| Multiple | 804 |
| Fracture of neck and trunk | 805-809 |
| Vertebral column without spinal cord | 805 |
| Vertebral column with spinal cord | 806 |
| Ribs, sternum, larynx, trachea | 807 |
| Pelvis | 808 |
| Unspecified | 809 |
| Fracture of upper limb | 810-819 |
| Fracture of lower limb | 820-829 |
| Dislocation | 830-839 |
| Sprains and strains of joints | 840-849 |
| Intracranial injury | 850-854 |
| Thorax, abdomen and pelvis | 860-869 |
| Open wound of head, neck and trunk | 870-879 |
| Ocular adnexa | 870 |
| Eyeball | 871 |
| Ear | 872 |
| Other head | 873 |
| Neck | 874 |
| Chest | 875 |
| Back | 876 |
| Buttocks | 877 |
| Genital organs | 878 |
| Unspecified | 879 |
| Open wound of upper limb | 880-887 |
| Open would of lower limb | 890-897 |
| Injury to blood vessels | 900-904 |
