# Supplementary material for: Pediatric patients with dog bites presenting to US children’s hospitals
Source: Inj Epidemiol. 2021 Sep 13;8:55. doi: 10.1186/s40621-021-00349-3 (PMC8436008; doi:10.1186/s40621-021-00349-3)
Supplement: Supplementary file 2 — Additional file 2: Table S2. Procedures, by groupings of International Classification of Disease, 9th revision (ICD-9) codes. [file 40621_2021_349_MOESM2_ESM.docx]

**Additional file 2: Table S2.** Procedures, by groupings of International Classification of Disease, 9^th^ revision (ICD-9) codes.

| **Procedure group** | **ICD-9 code** |
| --- | --- |
| Central nervous system | 01-05 |
| Eye | 08-16 |
| Ear | 18-20 |
| Nose, mouth and pharynx | 21-29 |
| Gastrointestinal | 42-54 |
| Genitourinary | 60-71 |
| Musculoskeletal | 76-84 |
| Integumentary | 85-86 |
