# Supplementary figures and images for: Pediatric patients with dog bites presenting to US children’s hospitals
Source: Inj Epidemiol. 2021 Sep 13;8:55. doi: 10.1186/s40621-021-00349-3 (PMC8436008; doi:10.1186/s40621-021-00349-3)

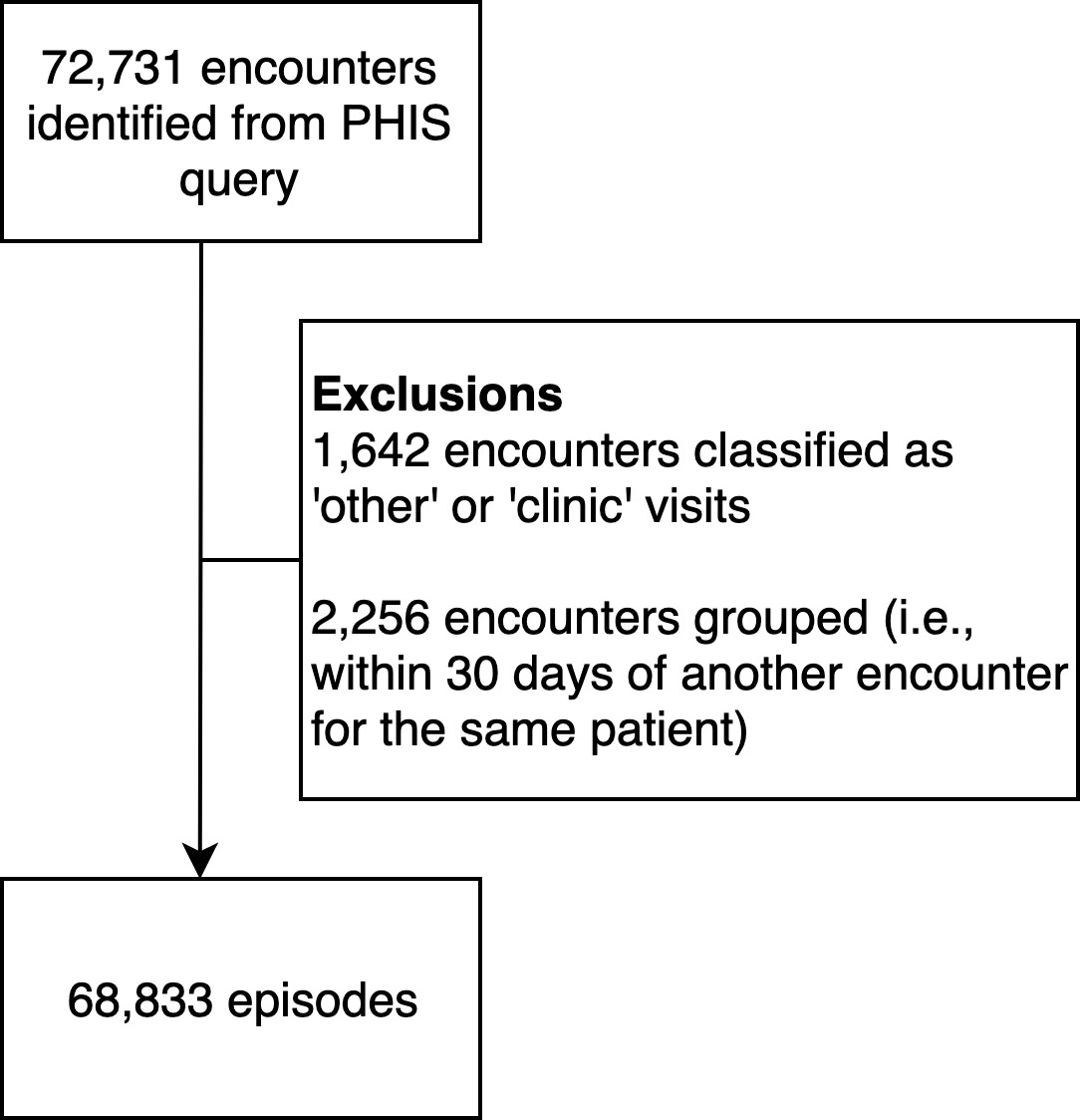

Supplement: Supplementary file 3 — Additional file 3: Figure. Patient inclusion. [file 40621_2021_349_MOESM3_ESM.jpg]
