# Supplementary material for: Pediatric patients with dog bites presenting to US children’s hospitals
Source: Inj Epidemiol. 2021 Sep 13;8:55. doi: 10.1186/s40621-021-00349-3 (PMC8436008; doi:10.1186/s40621-021-00349-3)
Supplement: Supplementary file 4 — Additional file 4: Table S3. Patient encounter pathways. [file 40621_2021_349_MOESM4_ESM.docx]

**Additional file 4: Table S3.** Patient encounter pathways

| **Encounter pathway** | **Number (column %)** | **Number requiring ICU (row %)** | **Diagnosis of skin/soft tissue infection (row %)** |
| --- | --- | --- | --- |
| **Single encounter within a 30-day range (n=66,752)** | | | |
| ED encounter only | 52,771 (79.1) | n/a | 1,024 (1.9) |
| ED encounter, use of OR/sedation | 6,757 (10.1) | n/a | 17 (0.3) |
| ED encounter, admitted; without use of OR/sedation | 1,960 (2.9) | 22 (1.1) | 1,453 (74.1) |
| ED encounter, admitted; with use of OR/sedation | 3,430 (5.1) | 169 (4.9) | 297 (8.7) |
| Admitted without ED encounter, without use of OR/sedation | 308 (0.3) | 5 (2.6) | 231 (75.0) |
| Admitted without ED encounter, with use OR/sedation | 216 (0.5) | 27 (12.5) | 44 (20.4) |
| Ambulatory surgery | 1,310 (2.0) | n/a | 13 (1.0) |
| **Multiple encounters in a 30-day range (n=2,081)** | | | |
| ED encounters only | 944 (45.4) | n/a | 102 (10.8) |
| ED encounter with use of OR/sedation | 165 (7.9) | n/a | 7 (4.2) |
| At least one admission without use of OR (inpatient or ambulatory surgery) or sedation | 406 (19.5) | 2 (0.5) | 351 (86.4) |
| At least one admission with use of OR (inpatient or ambulatory surgery) or sedation | 333 (1.6) | 17 (5.1) | 155 (46.5) |
| Ambulatory surgery only (with our without ED encounters) | 233 (11.2) | n/a | 14 (6.0) |

ED, emergency department; OR, operating room; ICU, intensive care unit
