# Supplementary material for: Pediatric patients with dog bites presenting to US children’s hospitals
Source: Inj Epidemiol. 2021 Sep 13;8:55. doi: 10.1186/s40621-021-00349-3 (PMC8436008; doi:10.1186/s40621-021-00349-3)
Supplement: Supplementary file 5 — Additional file 5: Table S4. Factors associated with clinically significant dog bite injuries as a sensitivity analysis when limited to encounters of patients ≤ 4 years of age (n = 26,162). [file 40621_2021_349_MOESM5_ESM.docx]

**Additional file 5: Table S4.** Factors associated with clinically significant dog bite injuries as a sensitivity analysis when limited to encounters of patients ≤4 years of age (n=26,162).

| **Variable** | **Not clinically significant (N=17,626)** | **Clinically significant (N=8,536)** | **Univariable odds of clinically significant injury** | | **Multivariable odds of clinically significant injury** | |
| --- | --- | --- | --- | --- | --- | --- |
|  | **N (%)** | **N (%)** | **OR (95% CI)** | **P** | **aOR (95% CI)** | **P** |
| Male sex | 9,429 (53.5) | 4,429 (51.9) | 0.94 (0.89-0.99) | 0.992 | 0.95 (0.90-1.01) | 0.077 |
| Race |  |  |  |  |  |  |
| White | 12,454 (70.7) | 6,822 (79.9) | Ref | -- | Ref | -- |
| Black | 2,317 (13.1) | 729 (8.5) | 0.50 (0.46-0.55) | <0.001 | 0.44 (0.39-0.48) | <0.001 |
| Other | 2,855 (16.2) | 985 (11.5) | 0.65 (0.60-0.71) | <0.001 | 0.74 (0.67-0.81) | <0.001 |
| Hispanic or Latino | 4,980 (28.3) | 1,631 (19.1) | 0.58 (0.54-0.62) | <0.001 | 0.53 (0.49-0.57) | <0.001 |
| Payor type |  |  |  |  |  |  |
| Public | 9,006 (51.1) | 4,374 (51.2) | Ref | -- | Ref | -- |
| Private | 7,031 (67.7) | 3,348 (32.3) | 0.98 (0.93-1.04) | 0.576 | 0.75 (0.70-0.80) | <0.001 |
| Other/Unknown | 1,589 (9.0) | 814 (9.5) | 0.95 (0.86-1.05) | 0.299 | 0.86 (0.78-0.94) | 0.002 |
| Weekday encounter | 11,433 (64.9) | 5,706 (66.8) | 1.09 (1.00-1.15) | 0.004 | 1.09 (1.03-1.15) | 0.004 |
| Season |  |  |  |  |  |  |
| Winter | 3,885 (22.0) | 1,971 (23.1) | Ref | -- | Ref | -- |
| Spring | 4,988 (28.3) | 2,333 (27.3) | 0.93 (0.86-1.00) | 0.052 | 0.94 (0.87-1.01) | 0.101 |
| Summer | 4,639 (26.3) | 2,178 (25.5) | 0.94 (0.87-1.01) | 0.100 | 0.93 (0.86-1.01) | 0.072 |
| Fall | 4,114 (23.3) | 2,054 (24.1) | 1.00 (0.92-1.08) | 0.928 | 0.99 (0.91-1.07) | 0.738 |
| Median household income, quartile |  |  |  |  |  |  |
| First | 3,826 (21.7) | 1,693 (19.8) | Ref | -- | Ref | -- |
| Second | 4,492 (25.5) | 2,262 (26.5) | 1.18 (1.09-1.28) | <0.001 | 1.06 (0.97-1.15) | 0.184 |
| Third | 4,760 (27.0) | 2,313 (27.1) | 1.26 (1.16-1.37) | <0.001 | 1.09 (1.00-1.19) | 0.044 |
| Fourth | 4,548 (25.8) | 2,268 (26.6) | 1.27 (1.17-1.39) | <0.001 | 1.01 (0.92-1.10) | 0.887 |

OR, odds ratio, aOR, adjusted odds ratio; CI, confidence interval
