# Supplementary material for: Pediatric patients with dog bites presenting to US children’s hospitals
Source: Inj Epidemiol. 2021 Sep 13;8:55. doi: 10.1186/s40621-021-00349-3 (PMC8436008; doi:10.1186/s40621-021-00349-3)
Supplement: Supplementary file 6 — Additional file 6: Table S5. Exploratory analysis of factors associated with clinically important outcomes, analyzed by individual outcome measures; outcome 1: admission. [file 40621_2021_349_MOESM6_ESM.docx]

**Additional file 6: Table S5.** Exploratory analysis of factors associated with clinically important outcomes, analyzed by individual outcome measures; outcome 1: admission

| **Variable** | **Not admitted (N=62,180)** | **Admitted (N-6,653)** | **Univariable odds of admission** | | **Multivariable odds of admission** | |
| --- | --- | --- | --- | --- | --- | --- |
|  | **N (%)** | **N (%)** | **OR (95% CI)** | **P** | **aOR (95% CI)** | **P** |
| Age |  |  |  |  |  |  |
| 0-4 years | 22,967 (36.9) | 3,195 (48.0) | 1.81 (1.61-2.05) | <0.001 | 1.59 (1.41-1.79) | <0.001 |
| 5-9 years | 21,688 (34.9) | 2031 (30.5) | 1.23 (1.09-1.39) | 0.001 | 1.16 (1.02-1.31) | 0.023 |
| 10 to 14 years | 13,336 (21.4) | 1,109 (16.7) | 1.09 (0.95-1.24) | 0.215 | 1.05 (0.92-1.20) | 0.474 |
| 15-18 years | 4,189 (6.7) | 318 (4.8) | Ref | -- | Ref | -- |
| Male sex | 34,514 (55.5) | 3,681 (55.3) | 1.00 (0.95-1.05) | 0.888 | 1.03 (0.98-1.09) | 0.228 |
| Race |  |  |  |  |  |  |
| White | 40,813 (65.6) | 4,973 (74.7) | Ref | -- | Ref | -- |
| Black | 11,562 (18.6) | 972 (14.6) | 0.60 (0.56-0.65) | 0.001 | 0.56 (0.51-0.60) | <0.001 |
| Other | 9,805 (15.8) | 708 (10.6) | 0.66 (0.60-0.72) | 0.001 | 0.74 (0.68-0.81) | <0.001 |
| Hispanic or Latino | 17,875 (28.7) | 1,296 (19.5) | 0.57 (0.53-0.61) | 0.001 | 0.52 (0.48-0.56) | <0.001 |
| Payor type |  |  |  |  |  |  |
| Public | 32,971 (53.0) | 3,678 (55.3) | Ref | -- | Ref | -- |
| Private | 23,025 (37.0) | 2,438 (36.6) | 0.69 (0.63-0.76) | 0.056 | 0.73 (0.69-0.78) | <0.001 |
| Other/Unknown | 6,184 (9.9) | 537 (8.1) | 0.95 (0.90-1.00) | 0.001 | 0.64 (0.58-0.71) | <0.001 |
| Weekday encounter | 40,276 (64.8) | 4,539 (68.2) | 1.20 (1.10-1.23) | 0.001 | 1.16 (1.10-1.23) | <0.001 |
| Season |  |  |  |  |  |  |
| Winter | 13,534 (21.8) | 1,503 (22.6) | Ref | -- | Ref | -- |
| Spring | 18,075 (29.1) | 1,928 (29.0) | 0.97 (0.91-1.05) | 0.489 | 0.99 (0.92-1.06) | 0.759 |
| Summer | 16,922 (27.2) | 1,735 (26.1) | 0.95 (0.88-1.02) | 0.132 | 0.95 (0.88-1.02) | 0.165 |
| Fall | 13,649 (22.0) | 1,487 (22.4) | 0.98 (0.91-1.06) | 0.653 | 0.97 (0.90-1.05) | 0.415 |
| Median household income, quartile |  |  |  |  |  |  |
| First | 15806 (25.4) | 1449 (21.8) | Ref | -- | Ref | -- |
| Second | 15375 (24.7) | 1863 (28.0) | 1.34 (1.24-1.44) | 0.001 | 1.20 (1.11-1.29) | <0.001 |
| Third | 15443 (24.8) | 1737 (26.1) | 1.36 (1.26-1.47) | 0.001 | 1.18 (1.08-1.27) | <0.001 |
| Fourth | 15556 (25.0) | 1604 (24.1) | 1.25 (1.15-1.35) | 0.001 | 0.99 (0.91-1.09) | 0.908 |

OR, odds ratio, aOR, adjusted odds ratio; CI, confidence interval
