# Supplementary material for: Pediatric patients with dog bites presenting to US children’s hospitals
Source: Inj Epidemiol. 2021 Sep 13;8:55. doi: 10.1186/s40621-021-00349-3 (PMC8436008; doi:10.1186/s40621-021-00349-3)
Supplement: Supplementary file 7 — Additional file 7: Table S6. Exploratory analysis of factors associated with clinically important outcomes, analyzed by individual outcome measures; outcome 2: sedation or operating room charge. [file 40621_2021_349_MOESM7_ESM.docx]

**Additional file 7: Table S6.** Exploratory analysis of factors associated with clinically important outcomes, analyzed by individual outcome measures; outcome 2: sedation or operating room charge

| **Variable** | **No use of sedation or operating room (N=** **56,441)** | **Use of sedation or operating room (n=12,392)** | **Univariable odds of sedation/operating room** | | **Multivariable odds of sedation/operating room** | |
| --- | --- | --- | --- | --- | --- | --- |
|  | **N (%)** | **N (%)** | **OR (95% CI)** | **P** | **OR (95% CI)** | **P** |
| Age |  |  |  |  |  |  |
| 0-4 years | 31,618 (56.0) | 6,577 (53.1) | 7.67 (6.63-8.88) | <0.001 | 6.67 (5.76-7.72) | <0.001 |
| 5-9 years | 19,455 (34.5) | 6,707 (54.1) | 4.61 (3.98-5.32) | <0.001 | 4.35 (3.76-5.04) | <0.001 |
| 10 to 14 years | 19,662 (34.8) | 4,057 (32.7) | 2.40 (2.06-2.80) | <0.001 | 2.33 (2.00-2.72) | <0.001 |
| 15-18 years | 13,017 (23.1) | 1,428 (11.5) | Ref | -- | Ref | -- |
| Male sex | 4,307 (7.6) | 200 (1.6) | 0.89 (0.86-0.93) | <0.001 | 0.95 (0.91-0.99) | 0.008 |
| Race |  |  |  |  |  |  |
| White | 36,218 (64.2) | 9,568 (77.2) | Ref | -- | Ref | -- |
| Black | 11,070 (19.6) | 1,464 (11.8) | 0.46 (0.43-0.48) | <0.001 | 0.47 (0.44-0.50) | <0.001 |
| Other | 9,153 (16.2) | 1,360 (11.0) | 0.57 (0.53-0.61) | <0.001 | 0.69 (0.65-0.74) | <0.001 |
| Hispanic or Latino | 16,765 (29.7) | 2,406 (19.4) | 0.51 (0.48-0.53) | <0.001 | 0.48 (0.45-0.51) | <0.001 |
| Payor type |  |  |  |  |  |  |
| Public | 30,261 (53.6) | 6,388 (51.5) | Ref | -- | Ref | -- |
| Private | 20,708 (36.7) | 4,755 (38.4) | 1.11 (1.06-1.16) | <0.001 | 0.73 (0.69-0.78) | <0.001 |
| Other/Unknown | 5,472 (9.7) | 1,249 (10.1) | 0.95 (0.89-1.02) | 0.186 | 0.81 (0.77-0.85) | <0.001 |
| Weekday encounter | 36,817 (65.2) | 7,998 (64.5) | 0.97 (0.93-1.01) | 0.094 | 0.98 (0.94-1.02) | 0.263 |
| Season |  |  |  |  |  |  |
| Winter | 12,268 (21.7) | 2,769 (22.3) | Ref | -- | Ref | -- |
| Spring | 16,506 (29.2) | 3,497 (28.2) | 0.96 (0.90-1.01) | 0.116 | 0.98 (0.92-1.03) | 0.426 |
| Summer | 15,381 (27.3) | 3,276 (26.4) | 0.97 (0.92-1.03) | 0.354 | 0.98 (0.93-1.04) | 0.576 |
| Fall | 12,286 (21.8) | 2,850 (23.0) | 1.03 (0.98-1.10) | 0.254 | 1.02 (0.96-1.08) | 0.592 |
| Median household income, quartile |  |  |  |  |  |  |
| First | 14,589 (25.8) | 2,666 (21.5) | Ref | -- | Ref |  |
| Second | 13,866 (24.6) | 3,372 (27.2) | 1.37 (1.29-1.45) | <0.001 | 1.12 (1.06-1.19) | <0.001 |
| Third | 13,948 (24.7) | 3,232 (26.1) | 1.44 (1.36-1.53) | <0.001 | 1.09 (1.03-1.17) | 0.006 |
| Fourth | 14,038 (24.9) | 3,122 (25.2) | 1.39 (1.31-1.48) | <0.001 | 0.95 (0.88-1.01) | 0.106 |

OR, odds ratio, aOR, adjusted odds ratio; CI, confidence interval
