# Supplementary material for: Pediatric patients with dog bites presenting to US children’s hospitals
Source: Inj Epidemiol. 2021 Sep 13;8:55. doi: 10.1186/s40621-021-00349-3 (PMC8436008; doi:10.1186/s40621-021-00349-3)
Supplement: Supplementary file 8 — Additional file 8: Table S7. Exploratory analysis of factors associated with clinically important outcomes, analyzed by individual outcome measures; outcome 3: fractures (excluding skull fractures). [file 40621_2021_349_MOESM8_ESM.docx]

**Additional file 8: Table S7.** Exploratory analysis of factors associated with clinically important outcomes, analyzed by individual outcome measures; outcome 3: fractures (excluding skull fractures)

| **Variable** | **No fractures (N=** **68,356)** | **Fracture present (n=** **477)** | **Univariable odds of fracture** | | **Multivariable odds of fracture** | |
| --- | --- | --- | --- | --- | --- | --- |
|  | **N (%)** | **N (%)** | **OR (95% CI)** | **P** | **aOR (95% CI)** | **P** |
| Age |  |  |  |  |  |  |
| 0-4 years | 26,038 (38.1) | 124 (26.0) | 0.50 (0.35-0.72) | <0.001 | 0.47 (0.33-0.67) | <0.001 |
| 5-9 years | 23,577 (34.5) | 142 (29.8) | 0.64 (0.45-0.90) | <0.001 | 0.63 (0.44-0.89) | 0.008 |
| 10 to 14 years | 14,276 (20.9) | 169 (35.4) | 1.25 (0.89-1.76) | <0.001 | 1.24 (0.88-1.75) | 0.209 |
| 15-18 years | 4,465 (6.5) | 42 (8.8) | Ref | -- | Ref | -- |
| Male sex | 37,917 (55.5) | 278 (58.3) | 1.12 (0.93-1.34) | 0.225 | 1.08 (0.90-1.30) | 0.415 |
| Race |  |  |  |  |  |  |
| White | 45,454 (66.5) | 332 (69.6) | Ref | -- | Ref | -- |
| Black | 12,448 (18.2) | 86 (18.0) | 0.92 (0.72-1.17) | 0.489 | 0.61 (0.46-0.79) | <0.001 |
| Other | 10,454 (15.3) | 59 (12.4) | 0.81 (0.61-1.08) | 0.146 | 0.78 (0.58-1.05) | 0.100 |
| Hispanic or Latino | 19,057 (27.9) | 114 (23.9) | 0.82 (0.66-1.02) | 0.080 | 0.64 (0.50-0.82) | <0.001 |
| Payor type |  |  |  |  |  |  |
| Public | 36,359 (53.2) | 290 (60.8) | Ref | -- | Ref | -- |
| Private | 25,313 (37.0) | 150 (31.4) | 0.75 (0.61-0.92) | 0.005 | 0.71 (0.57-0.89) | 0.002 |
| Other/Unknown | 6,684 (9.8) | 37 (7.8) | 0.68 (0.48-0.95) | 0.025 | 0.65 (0.46-0.92) | 0.015 |
| Weekday encounter | 44,485 (65.1) | 330 (69.2) | 1.20 (0.99-1.46) | 0.066 | 1.18 (0.97-1.43) | 0.347 |
| Season |  |  |  |  |  |  |
| Winter | 14,932 (21.8) | 105 (22.0) | Ref | -- | Ref | -- |
| Spring | 19,880 (29.1) | 123 (25.8) | 0.89 (0.68-1.15) | 0.361 | 0.88 (0.68-1.15) | 0.347 |
| Summer | 18,532 (27.1) | 125 (26.2) | 0.97 (0.74-1.26) | 0.817 | 0.96 (0.74-1.24) | 0.745 |
| Fall | 15,012 (22.0) | 124 (26.0) | 1.18 (0.91-1.53) | 0.216 | 1.18 (0.91-1.53) | 0.216 |
| Median household income, quartile |  |  |  |  |  |  |
| First | 17,116 (25.0) | 139 (29.1) | Ref | -- | Ref | -- |
| Second | 17,104 (25.0) | 134 (28.1) | 0.97 (0.76-1.23) | 0.799 | 0.97 (0.76-1.24) | 0.805 |
| Third | 17,059 (25.0) | 121 (25.4) | 0.88 (0.68-1.13) | 0.309 | 0.89 (0.69-1.16) | 0.398 |
| Fourth | 17,077 (25.0) | 83 (17.4) | 0.61 (0.46-0.81) | <0.001 | 0.59 (0.44-0.80) | <0.001 |

OR, odds ratio, aOR, adjusted odds ratio; CI, confidence interval
