# Supplementary material for: Pediatric patients with dog bites presenting to US children’s hospitals
Source: Inj Epidemiol. 2021 Sep 13;8:55. doi: 10.1186/s40621-021-00349-3 (PMC8436008; doi:10.1186/s40621-021-00349-3)
Supplement: Supplementary file 9 — Additional file 9: Table S8. Exploratory analysis of factors associated with clinically important outcomes, analyzed by individual outcome measures; outcome 4: skull fractures, intracranial/orbital injury. [file 40621_2021_349_MOESM9_ESM.docx]

**Additional file 9: Table S8.** Exploratory analysis of factors associated with clinically important outcomes, analyzed by individual outcome measures; outcome 4: skull fractures, intracranial/orbital injury

| **Variable** | **No skull fracture, intracranial or orbital injury (N=** **68,144)** | **Skull fracture, intracranial or orbital injury (n=** **689)** | **Univariable odds of skull fracture, intracranial, orbital injury** | | **Multivariable odds of skull fracture, intracranial, orbital injury** | |
| --- | --- | --- | --- | --- | --- | --- |
|  | **N (%)** | **N (%)** | **OR (95% CI)** | **P** | **aOR (95% CI)** | **P** |
| Age |  |  |  |  |  |  |
| 0-4 years | 25,714 (37.7) | 448 (65.0) | 2.95 (1.98-4.39) | <0.001 | 2.52 (1.69-3.75) | <0.001 |
| 5-9 years | 23,569 (34.6) | 150 (21.8) | 1.08 (0.71-1.64) | 0.703 | 1.01 (0.67-1.54) | 0.956 |
| 10 to 14 years | 14,380 (21.1) | 65 (9.4) | 0.77 (0.49-1.21) | 0.257 | 0.74 (0.47-1.17) | 0.204 |
| 15-18 years | 4,481 (6.6) | 26 (3.8) | Ref | -- | Ref | -- |
| Male sex | 37,833 (55.5) | 362 (52.5) | 0.89 (0.76-1.03) | 0.113 | 0.94 (0.81-1.10) | 0.450 |
| Race |  |  |  |  |  |  |
| White | 45,238 (66.4) | 548 (79.5) | Ref | -- | Ref | -- |
| Black | 12,458 (18.3) | 76 (11.0) | 0.43 (0.34-0.56) | <0.001 | 0.45 (0.34-0.58) | <0.001 |
| Other | 10,448 (15.3) | 65 (9.4) | 0.55 (0.42-0.73) | <0.001 | 0.67 (0.51-0.88) | 0.004 |
| Hispanic or Latino | 19,051 (28.0) | 120 (17.4) | 0.51 (0.41-0.63) | <0.001 | 0.47 (0.38-0.60) | <0.001 |
| Payor type |  |  |  |  |  |  |
| Public | 36,274 (53.2) | 375 (54.4) | Ref | -- | Ref | -- |
| Private | 25,207 (37.0) | 256 (37.2) | 1.04 (0.88-1.22) | 0.668 | 0.80 (0.67-0.95) | 0.019 |
| Other/Unknown | 6,663 (9.8) | 58 (8.4) | 0.74 (0.57-0.99) | 0.049 | 0.71 (0.53-0.94) | 0.013 |
| Weekday encounter | 44,350 (65.1) | 465 (67.5) | 1.11 (0.94-1.30) | 0.215 | 1.10 (0.94-1.29) | 0.245 |
| Season |  |  |  |  |  |  |
| Winter | 14,883 (21.8) | 154 (22.4) | Ref | -- | Ref | -- |
| Spring | 19,803 (29.1) | 200 (29.0) | 1.00 (0.81-1.23) | 0.984 | 1.03 (0.83-1.28) | 0.778 |
| Summer | 18,483 (27.1) | 174 (25.3) | 0.94 (0.76-1.17) | 0.600 | 0.96 (0.77-1.19) | 0.696 |
| Fall | 14,975 (22.0) | 161 (23.4) | 1.05 (0.84-1.31) | 0.664 | 1.02 (0.82-1.28) | 0.859 |
| Median household income, quartile |  |  |  |  |  |  |
| First | 17,098 (25.1) | 157 (22.8) | Ref | -- | Ref | -- |
| Second | 17,025 (25.0) | 213 (30.9) | 1.39 (1.12-1.71) | 0.003 | 1.13 (0.91-1.40) | 0.266 |
| Third | 17,008 (25.0) | 172 (25.0) | 1.25 (0.99-1.56) | 0.057 | 0.94 (0.75-1.19) | 0.621 |
| Fourth | 17,013 (25.0) | 147 (21.3) | 1.10 (0.86-1.40) | 0.443 | 0.75 (0.58-0.97) | 0.030 |

OR, odds ratio, aOR, adjusted odds ratio; CI, confidence interval
