# Supplementary material for: Pediatric patients with dog bites presenting to US children’s hospitals
Source: Inj Epidemiol. 2021 Sep 13;8:55. doi: 10.1186/s40621-021-00349-3 (PMC8436008; doi:10.1186/s40621-021-00349-3)
Supplement: Supplementary file 10 — Additional file 10: Table S9. Exploratory analysis of factors associated with clinically important outcomes, analyzed by individual outcome measures; outcome 5: skin/soft tissue infection. [file 40621_2021_349_MOESM10_ESM.docx]

**Additional file 10: Table S9.** Exploratory analysis of factors associated with clinically important outcomes, analyzed by individual outcome measures; outcome 5: skin/soft tissue infection

| **Variable** | **No skin/soft tissue infection (N=** **65,125)** | **Skin/soft tissue infection (n=** **3,708)** | **Univariable odds of skin/soft tissue infection** | | **Multivariable odds of skin/soft tissue infection** | |
| --- | --- | --- | --- | --- | --- | --- |
|  | **N (%)** | **N (%)** | **OR (95% CI)** | **P** | **aOR (95% CI)** | **P** |
| Age |  |  |  |  |  |  |
| 0-4 years | 24,407 (37.5) | 1,755 (47.3) | 1.87 (1.04-1.36) | 0.014 | 1.09 (0.95-1.26) | 0.206 |
| 5-9 years | 22,649 (34.8) | 1,070 (28.9) | 0.79 (0.68-0.91) | <0.001 | 0.76 (0.66-0.88) | <0.001 |
| 10 to 14 years | 13,813 (21.2) | 632 (17.0) | 0.77 (0.66-0.89) | <0.001 | 0.75 (0.65-0.88) | <0.001 |
| 15-18 years | 4,256 (6.5) | 251 (6.8) | Ref | -- | Ref | -- |
| Male sex | 632 (17.0) | 2,000 (53.9) | 0.94 (0.88-1.01) | 0.081 | 0.97 (0.91-1.04) | 0.425 |
| Race |  |  |  |  |  |  |
| White | 43,116 (66.2) | 2,670 (72.0) | Ref | -- | Ref | -- |
| Black | 12,014 (18.4) | 520 (14.0) | 0.69 (0.63-0.76) | <0.001 | 0.72 (0.64-0.80) | <0.001 |
| Other | 9,995 (15.3) | 518 (14.0) | 0.83 (0.75-0.92) | <0.001 | 0.93 (0.83-1.03) | 0.172 |
| Hispanic or Latino | 18,315 (28.1) | 856 (23.1) | 0.74 (0.68-0.80) | <0.001 | 0.73 (0.66-0.80) | <0.001 |
| Payor type |  |  |  |  |  |  |
| Public | 34,689 (53.3) | 1,960 (52.9) | Ref | -- | Ref | -- |
| Private | 24,001 (36.9) | 1,462 (39.4) | 1.05 (0.98-1.23) | 0.196 | 0.87 (0.81-0.94) | <0.001 |
| Other/Unknown | 6,435 (9.9) | 286 (7.7) | 0.75 (0.66-0.85) | <0.001 | 0.71 (0.63-0.81) | <0.001 |
| Weekday encounter | 42,191 (64.8) | 2,624 (70.8) | 1.32 (1.22-1.41) | <0.001 | 1.31 (1.22-1.41) | <0.001 |
| Season |  |  |  |  |  |  |
| Winter | 14,207 (21.8) | 830 (22.4) | Ref | -- | Ref | -- |
| Spring | 18,914 (29.0) | 1,089 (29.4) | 0.98 (0.89-1.08) | 0.678 | 0.99 (0.91-1.09) | 0.886 |
| Summer | 17,671 (27.1) | 986 (26.6) | 0.95 (0.86-1.04) | 0.262 | 0.95 (0.87-1.05) | 0.331 |
| Fall | 14,333 (22.0) | 803 (21.7) | 0.95 (0.86-1.05) | 0.336 | 0.94 (0.85-1.04) | 0.237 |
| Median household income, quartile |  |  |  |  |  |  |
| First | 16,511 (25.4) | 744 (20.1) | Ref | -- | Ref | -- |
| Second | 16,320 (25.1) | 918 (24.8) | 1.22 (1.11-1.36) | <0.001 | 1.14 (1.03-1.27) | 0.011 |
| Third | 16,152 (24.8) | 1,028 (27.7) | 1.43 (1.29-1.58) | <0.001 | 1.31 (1.18-1.45) | <0.001 |
| Fourth | 16,142 (24.8) | 1,018 (27.5) | 1.39 (1.25-1.54) | <0.001 | 1.22 (1.09-1.37) | <0.001 |

OR, odds ratio, aOR, adjusted odds ratio; CI, confidence interval
