# Supplementary material for: Pediatric patients with dog bites presenting to US children’s hospitals
Source: Inj Epidemiol. 2021 Sep 13;8:55. doi: 10.1186/s40621-021-00349-3 (PMC8436008; doi:10.1186/s40621-021-00349-3)
Supplement: Supplementary file 11 — Additional file 11: Table S10. Exploratory analysis of factors associated with clinically important outcomes, analyzed by individual outcome measures; outcome 6: death. [file 40621_2021_349_MOESM11_ESM.docx]

**Additional file 11: Table S10.** Exploratory analysis of factors associated with clinically important outcomes, analyzed by individual outcome measures; outcome 6: death

| **Variable** | **No in-hospital mortality (N=** **68828)** | **In-hospital mortality (n=** **5)** | **Univariable odds of in-hospital mortality** | |
| --- | --- | --- | --- | --- |
|  | **N (%)** | **N (%)** | **OR (95% CI)** | **P** |
| Age |  |  |  |  |
| 0-4 years | 26,158 (38.0) | 4 (80.0) | Inf (0-Inf) | 0.998 |
| 5-9 years | 23,718 (34.5) | 1 (20.0) | Inf (0-Inf) | 0.998 |
| 10 to 14 years | 14,445 (21.0) | 0 (0.0) | Inf (0-Inf) | 1.00 |
| 15-18 years | 4,507 (6.5) | 0 (0.0) | Ref | -- |
| Male sex | 38,191 (55.5) | 4 (80.0) | 3.20 (0.36-29) | 0.297 |
| Race |  |  |  |  |
| White | 45,784 (66.5) | 2 (40.0) | Ref | -- |
| Black | 12,532 (18.2) | 2 (40.0) | 3.70 (0.51-26) | 0.195 |
| Other | 10,512 (15.3) | 1 (20.0) | 2.20 (0.20-24) | 0.525 |
| Hispanic or Latino | 19,170 (27.9) | 1 (20.0) | 0.65 (0.07-5.79) | 0.698 |
| Payor type |  |  |  |  |
| Public | 36,646 (53.2) | 3 (60.0) | Ref | -- |
| Private | 25,463 (37.0) | 0 (0.0) | 0.00 (0.00-Inf) | 0.996 |
| Other/Unknown | 6,719 (9.8) | 2 (40.0) | 3.60 (0.61-22) | 0.157 |
| Weekday encounter | 44,812 (65.1) | 3 (60.0) | 0.80 (0.13-4.81) | 0.811 |
| Season |  |  |  |  |
| Winter | 15,035 (21.8) | 2 (40.0) | Ref | -- |
| Spring | 20,001 (29.1) | 2 (40.0) | 0.75 (0.11-5.33) | 0.775 |
| Summer | 18,656 (27.1) | 1 (20.0) | 0.40 (0.04-4.44) | 0.458 |
| Fall | 15,136 (22.0) | 0 (0.0) | 0.00 (0.00-Inf) | 0.995 |
| Median household income, quartile |  |  |  |  |
| First | 17253 (25.1) | 2 (40.0) | Ref | -- |
| Second | 17238 (25.0) | 0 (0.0) | 0.00 (0.00-Inf) | 0.995 |
| Third | 17178 (25.0) | 2 (40.0) | 1.00 (0.14-7.13) | 0.997 |
| Fourth | 17159 (24.9) | 1 (20.0) | 0.50 (0.05-5.54) | 0.574 |

OR, odds ratio, CI, confidence interval; Inf, infinity
